# A Personalized 4D Workflow for the Manufacturing of Functional and Removable Esthetic Devices: A Technical Report

**DOI:** 10.1111/jerd.13454

**Published:** 2025-03-03

**Authors:** Mauro Merli, Luca Aquilanti, Alessio Casucci, Michele Nieri, Giorgia Mariotti, Marco Merli, Giorgio Rappelli

**Affiliations:** ^1^ Department of Clinical Specialistic and Dental Sciences Polytechnic University of Marche Ancona Italy; ^2^ Clinica Merli Rimini Italy; ^3^ Department of Medical Biotechnologies University of Siena Siena Italy; ^4^ Department of Clinical and Experimental Medicine University of Florence Florence Italy; ^5^ Dentistry Clinic National Institute of Health and Science of Aging Ancona Italy

**Keywords:** 4D virtual patient, diagnostic esthetic device, jaw motion tracking, maxillomandibular relationship, occlusal vertical dimension, rest position

## Abstract

**Objective:**

This technical report describes a fully digital workflow for the manufacturing of a functional removable esthetic device (FRED) that addresses multiple clinical scenarios before prosthetic rehabilitation. The proposed method integrates advanced digital tools to achieve functional and esthetic outcomes while ensuring reversibility and patient comfort.

**Clinical Considerations:**

The workflow includes intraoral and facial scanning, jaw motion tracking, and CAD/CAM technology to create a 4D virtual patient. This allows for a precise recording of the maxillomandibular relationship and facilitates the determination of the comfort position to establish a new occlusal vertical dimension (OVD). FRED is a custom‐designed, removable, tooth‐colored device manufactured to provide temporary functional support, esthetic preview, catering to different clinical scenarios, including tooth wear, occlusal trauma, and periodontal conditions.

**Conclusions:**

This technique combines digital and traditional prosthetic principles into a patient‐centered, adaptable workflow, allowing precise, non‐invasive, and dynamic evaluation of OVD modifications. By integrating digital recording, facial scanning, and CAD/CAM fabrication, it enhances function, esthetics, and patient comfort.

**Clinical Significance:**

This technique highlights the synergy of digital innovation and prosthetic principles, enhancing diagnostic capabilities and treatment predictability. The patient‐centered design supports functional stability and esthetic outcomes, making it a valuable tool in modern prosthetic dentistry.

## 1 Introduction

Patients in need of extensive prosthodontic treatment may require an alteration in their occlusal vertical dimension (OVD) due to tooth wear, advanced periodontal disease, tooth loss, or changes that have occurred to existing prostheses over time [1]. Generally, this process involves determining the new OVD, taking into account both functionality and esthetics. Accurate registration of the maxillomandibular relationship is a fundamental step in prosthetic rehabilitation [2]. The determination of OVD involves several factors, including facial esthetics, the rest position, phonetics, and craniofacial landmarks. Additionally, a precise recording and the hinge axis position are crucial for successful prosthetic outcomes [3].

Traditional jaw relation transfer methods, such as facebow techniques and mechanical articulators, are often complex, time‐consuming, and heavily reliant on the clinician's experience [3]. Moreover, they can be imprecise and uncomfortable for the patients. With advancements in digital dentistry, fixed prosthetic rehabilitation workflows have become more streamlined and efficient. Tools such as intraoral scanners (IOS), facial scans, cone beam computed tomography (CBCT), jaw motion tracking systems (JTS), and virtual articulators enable the creation of 3D or 4D virtual dental patients [4]. These technologies offer enhanced visualization and diagnostic capabilities compared to traditional methods, simplifying the analysis of complex cases [5]. Moreover, they allow for a more accurate and individualized approach [6]. The integration of 4D technology adds a new time‐based dimension to static 3D models, simulating dynamic occlusal interactions [5, 7].

This paper presents the technique to manufacture a Functional Removable Esthetic Device (FRED). FRED may be applied in multiple clinical scenarios central to modern prosthetic dentistry: functional and phonetic testing, occlusal stability evaluation, and esthetic previsualization to aid in treatment planning. Additionally, it may be used to test patients' adaptation to the new OVD, offering a dynamic but reversible and non‐invasive solution for patients with tooth wear or extensive restorative needs. Serving as a temporary prosthesis, it may stabilize teeth affected by periodontal disease, reducing occlusal trauma. Its diagnostic value supports complex cases like full‐mouth rehabilitation by integrating mandibular dynamics with craniofacial relationships through digital tools [8].

## 2 Technique

### 2.1 Patient Assessment and Data Collection

The clinical workflow begins with a comprehensive clinical and bi‐dimensional radiographic evaluation, which includes a detailed occlusal examination with attention to esthetic elements such as tooth alignment, smile symmetry, and overall facial appearance. A high‐resolution intraoral scanner (IOS) (Trios 5, 3Shape, Copenhagen, Denmark) is used to capture digital impressions of the upper and lower arches. This step ensures precise recording of occlusal surfaces and gingival contours, essential for accurate splint fitting. Additionally, any esthetic concerns expressed by the patient are carefully considered during FRED's design.

### 2.2 Digital Recording of Mandible Movement

The process for recording mandibular movement starts with the identification of the temporomandibular joint (TMJ) and the right infraorbital margin using the jaw tracking system (JTS; Cyclops, Itaka Way Med, Marcon, Italy). The JTS consists of two main components: hardware and software. The hardware system includes a capture source equipped with stereo‐vision cameras and several sensor‐integrated accessories, utilizing stereo‐photometry technology for precise mandibular motion tracking, as detailed below:

- A lower fork adapted to the mandibular teeth with bis‐acrylic resin (Acrytemp, Zhermack, Badia Polesine, Italy), serves as the mandibular tracker.
- An upper positioner fixed to the subject's head as a head frame.
- A fork adapted to the maxillary teeth using polyvinylsiloxane material (Hydrorise Medium Body, Zhermack, Badia Polesine, Italy) for maxillary position recording.
- A magnetic positioning device attached to the lower and upper fork, as well as to the upper positioner.

The stability of the lower fork and absence of occlusal interferences during mandibular movements are carefully verified.

The JTS records dynamic mandibular movements to design the FRED. Having placed the patient in a relaxed and upright position, the recording process includes a range of movements, such as open‐close, gothic arch tracing, protrusion, lateral excursions, comfort position, grinding, and chewing with and without bolus. For patients showing signs or symptoms of temporomandibular disorders (TMD) or requiring complex rehabilitation, a low‐dose cone‐beam computed tomography (CBCT) with a 16 × 16 field of view may be recommended to include TMJ landmarks and cranial reference points like the glabella or crista galli. Conducting the CBCT with a closed bite permits the evaluation of the condyle position within the articular fossae. Upon completing the recordings, the extent of the OVD increase is determined by analyzing the recorded kinematic motion data combined with intraoral scans. In particular, the process relies on identifying a mandibular position that integrates the comfort position of the jaw with the adequate prosthetic space. By examining the mandibular movement trajectories with the spatial position of the upper and lower arches and TMJ, the appropriate OVD change is determined. The recorded rest position is used as the new OVD in determining the maxilla‐mandibular position. Adjustments are made within the interocclusal rest space, balancing functional prosthetic requirements with esthetic goals, with the appropriate space distributed between dental arches.

Examples of deprogramming devices that are typically used in the recording process include Lucia Jigs, leaf gauges, and Kois deprogrammers. However, no such devices were used in this study.

### 2.3 Upper Fork Scan and 3D Facial Scan

The upper fork scan is performed to align dental arches with craniofacial structures, a key step for ensuring accurate occlusal plane orientation, particularly in planning temporary prostheses or esthetic visualization. A 3D facial scan (Cyclops, Itaka Way Med, Marcon, Italy) is then captured from multiple angles, with and without the upper fork, to assess facial proportions and integrate the smile with the overall facial structure. The 3D scan also shows the patient's smile at varying lip exposures, aiding to analyze FRED's esthetic impact in social settings, such as smiling.

### 2.4 CAD/CAM Phase

Digital scans, facial images, CBCT data (when applicable), and mandibular movement records are imported into CAD software (Exocad, exocad GmbH, Darmstadt, Germany) to create a 4D virtual model of the patient. The process begins with a digital wax‐up, in which tooth forms are selected based on the gothic arch pathway. Teeth are chosen from the CAD software library with cusps' width and inclination that match the arch's path.

Using anatomical guidelines derived from CBCT data (or JTS references for TMJ and infraorbital alignment), tools such as Monson's sphere are used to orient the occlusal plane [9]. The sphere's center aligns with the crista galli apophysis, while its periphery is positioned along the condylar borders. Then, considering the recorded jaw movements, teeth are rotated by a few degrees and/or morphologically modified to eliminate any interferences and establish correct contacts. The esthetic parameters are guided by the patient's facial proportions, smile dynamics, and specific esthetic preferences discussed during the clinical evaluation. The position of the incisal edges of the upper incisors are determined considering patient's lip position and smile dynamics, ensuring that the incisal edges would be harmoniously positioned for functional and esthetic outcomes.

The software is able to remove undercuts to optimize mechanical retention and establish FRED's path of insertion. Through the Boolean subtraction function, the software is able to remove from the digital waxing patient's teeth. The final modeling product is ready to be milled. The CAD design is exported to CAM software (inLab CAM 19.0, Dentsply Sirona, Charlotte, USA). A multilayer shaded polymethyl methacrylate (PMMA) disc, with high flexural strength and high esthetic characteristics, is selected (Multilayer PMMA Discs—A2—98.5 × 20 mm, Dentsply Sirona, Charlotte, USA). The milling strategy of the dental milling machine (inLab MC X5, Dentsply Sirona, Charlotte, USA) is set, selecting the one with the highest degree of detail level. Milling takes 6–8 h, followed by polishing and fine adjustments for a precise fit.

### 2.5 Splint Fitting and Patient Evaluation

FRED is fitted in the patient's mouth, utilizing natural tooth undercuts to retain it. Initially, patients may feel interferences as the comfort position typically shifts forward from maximum intercuspation. Occlusal balance across various jaw movements is assessed, and the splint's impact on the patient's smile is verified to ensure a natural appearance.

### 2.6 Patient Follow‐Up for Functional, Esthetic, and Prosthetic Evaluation

Patients receive instructions for splint care and usage, with follow‐up appointments scheduled just a few days later to evaluate occlusal rebalancing and muscle relaxation. Esthetic satisfaction is reviewed, and adjustments are made if needed. For patients using FRED as a temporary prosthesis, plans are made for definitive restoration based on the FRED's results. Jaw motion tracking may be repeated if necessary to monitor functional progress.

## 3 Clinical Case

**FIGURE 1:**
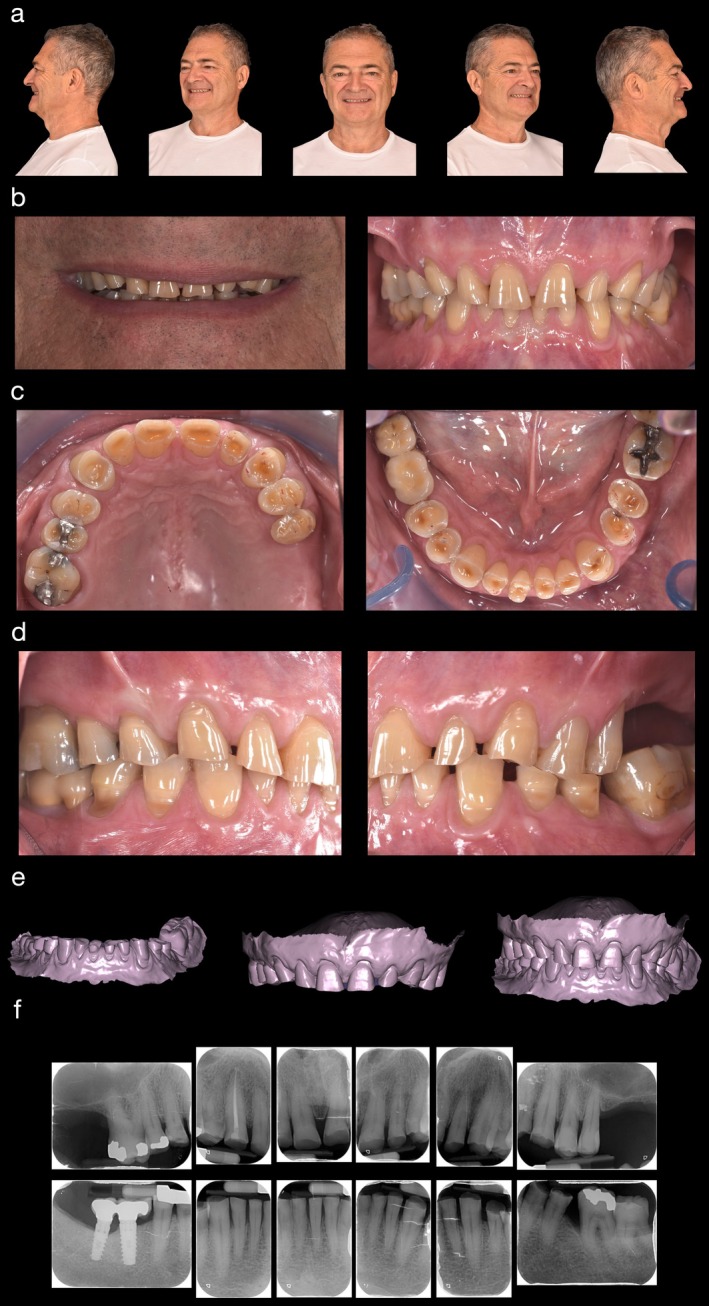
(a–f) Patient assessment and data collection: (a) extraoral images (smiling and not smiling), (b–d) smile detail and intraoral images (frontal, occlusal, lateral), (e) intraoral scan, (f) 2D radiographic images.

A 67‐year‐old Caucasian male presented with concerns about the esthetic appearance of his anterior teeth and defects in his posterior teeth. Clinical examination revealed severe wear and dentin exposure across the entire dentition (Figure 1a–f), with a Basic Erosive Wear Examination (BEWE) score of 18 [10]. The patient reported no TMD symptoms. TMJ and muscle palpation indicated no pain, and occlusal assessment showed that the anterior teeth lacked sufficient guidance to disarticulate the posterior teeth during protrusive movements.

**FIGURE 2:**
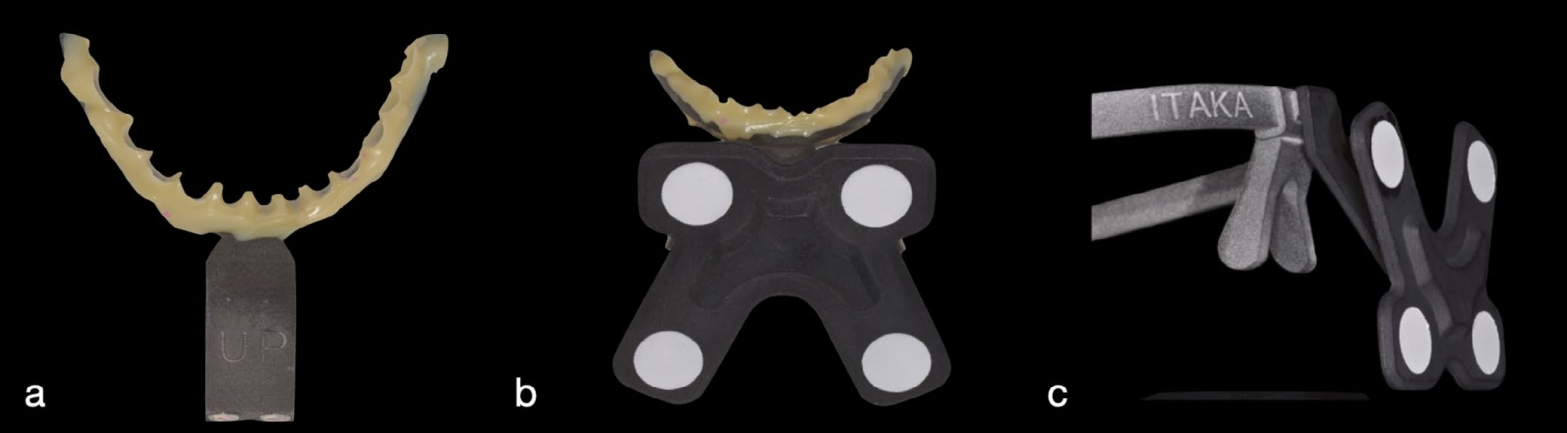
(a) The lower fork adapted to the mandibular teeth with bis‐acrylic resin. (b) The magnetic positioning device attached to the lower fork. (c) The upper positioner fixed to the subject's head as a head frame.

**FIGURE 3:**
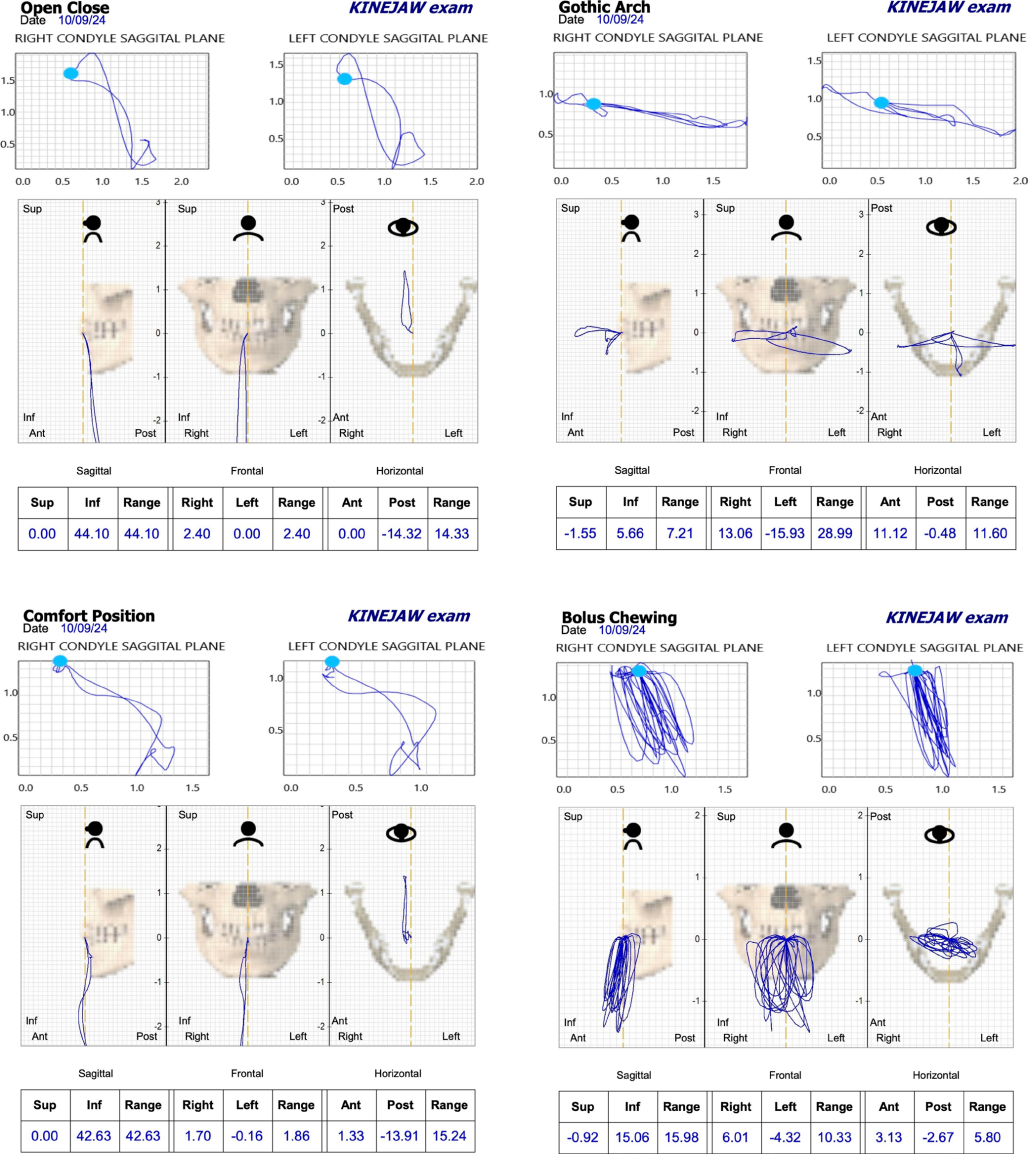
Jaw motion tracking using Itaka (Cyclops, Itaka Way Med, Marcon, Italy). In particular, the figure shows the envelop of the open‐close, gothic arch, comfort position and bolus chewing movements.

**FIGURE 4:**
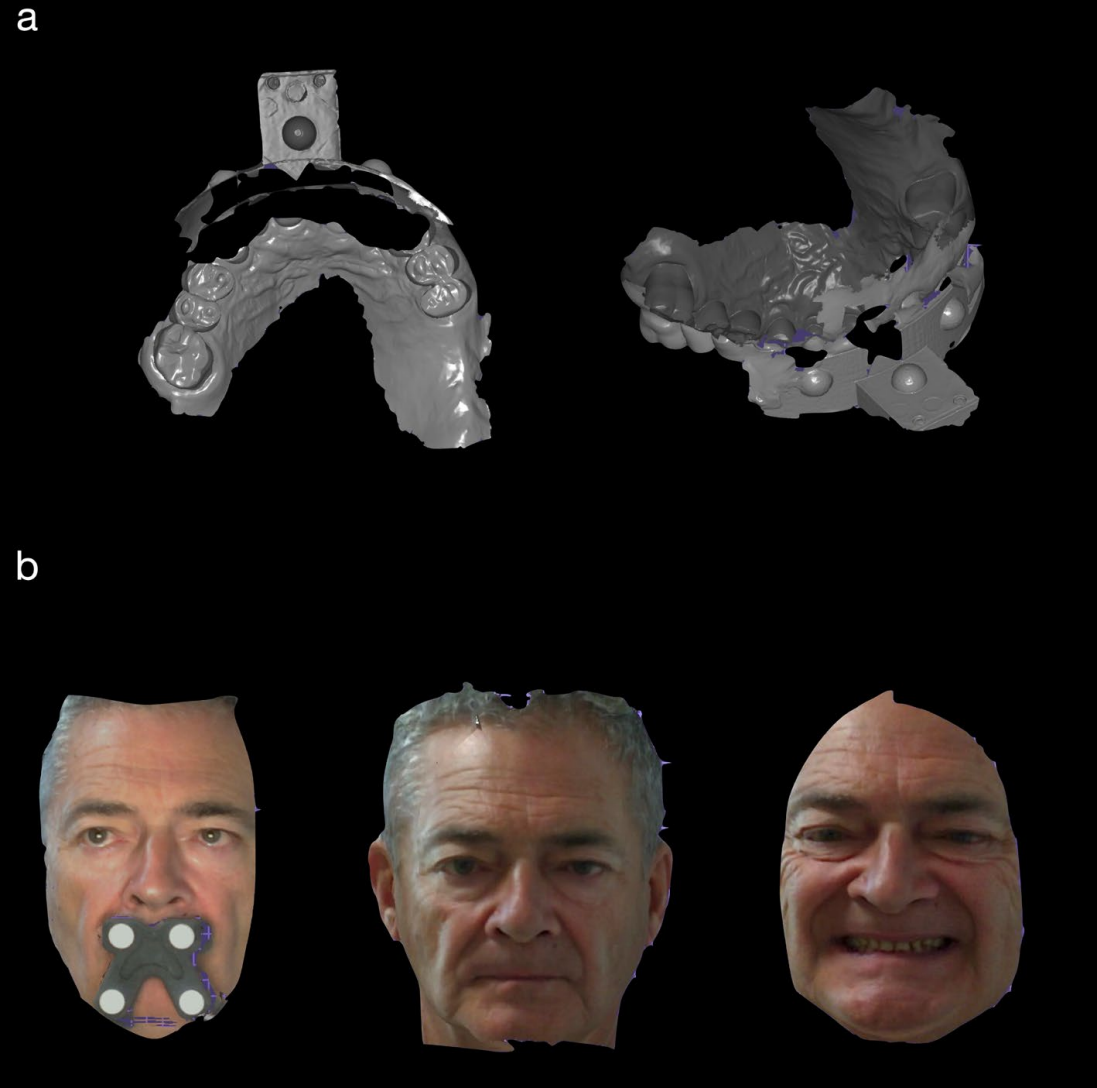
(a) Upper fork scan, (b) 3D face scan showing esthetic analysis with varied smile exposures, with and without the upper fork.

**FIGURE 5:**
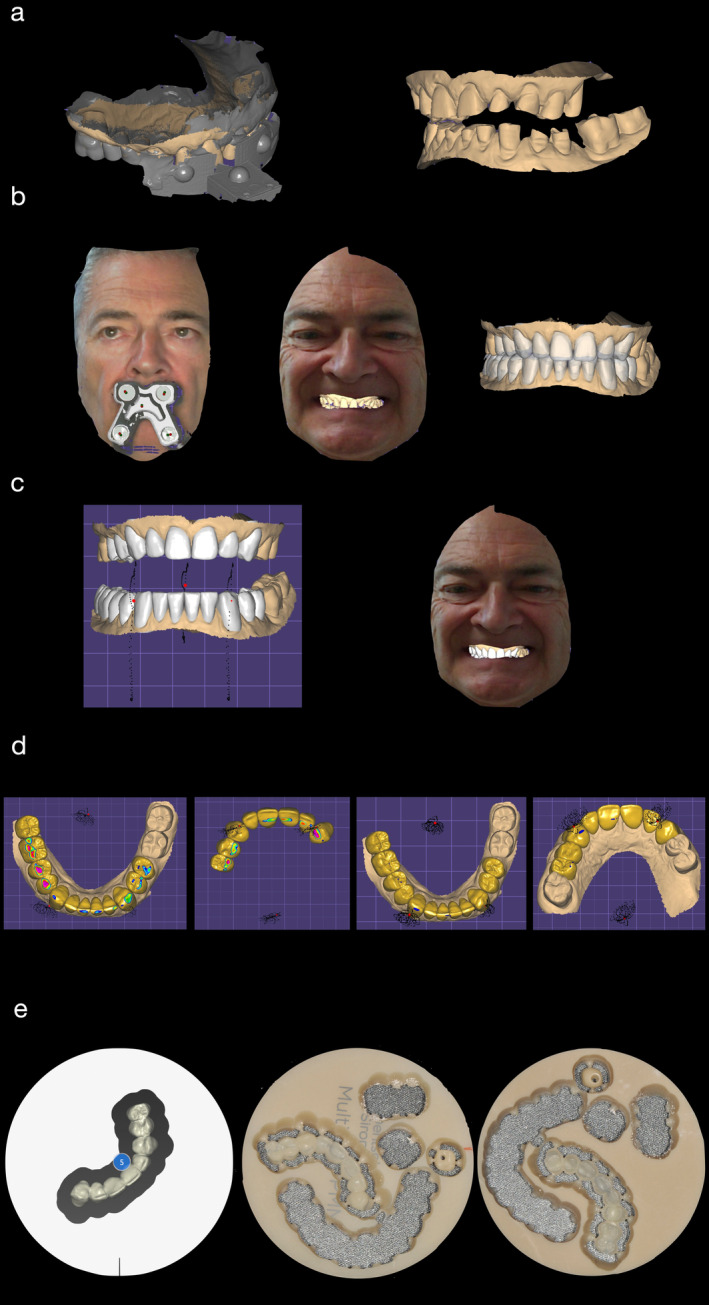
(a–d) CAD design showing integration of functional and esthetic elements in the FRED (Exocad, exocad GmbH, Darmstadt, Germany). (e) Industrial prefabricated CAD/CAM disc made of tooth‐colored polymethylmethacrylate (PMMA) (Multilayer PMMA Discs, Dentsply Sirona, Charlotte, USA) after the milling procedure (inLab MC X5, Dentsply Sirona, Charlotte, USA).

**FIGURE 6:**
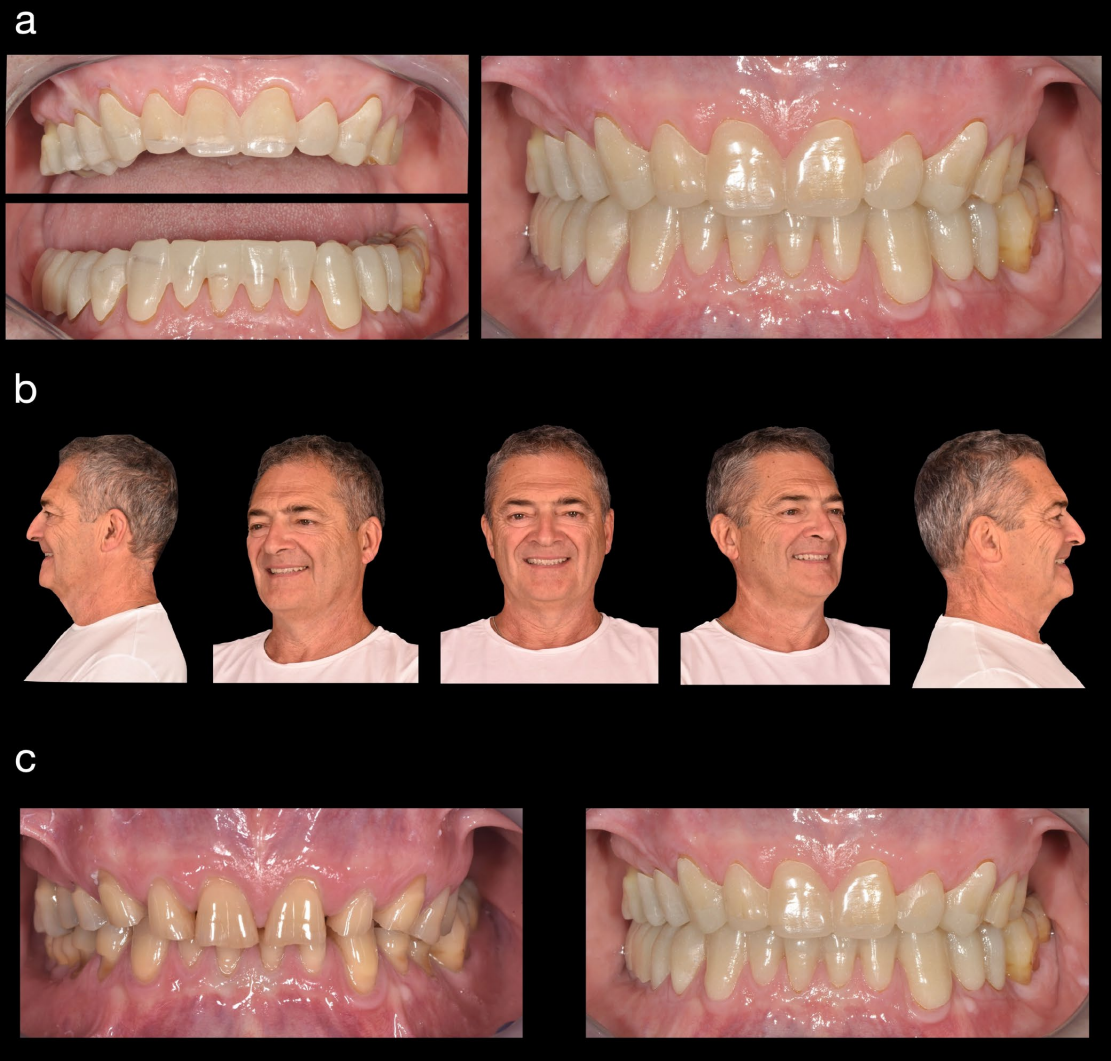
(a–c) Patient wearing the FRED with a balanced occlusal plane and natural smile. Vertical occlusal dimension was increased by 3.2 mm. (c) Pre‐ and post‐op comparison.

Following the outlined digital workflow, data collection and assessment were initiated to guide the treatment plan. The patient's mandibular rest position was recorded, and an appropriate OVD was determined. This process integrated space requirements for restoration with the esthetic objectives for the case, using comprehensive intraoral and facial scanning in conjunction with jaw motion tracking. The data from these scans were used in CAD/CAM processes to create diagnostic, tooth‐colored functional splints designed to enhance esthetic and functional outcomes (Figures 2a–c, 3, 4, 5a–e, and 6a–c).

Two custom‐designed, tooth‐colored splints were fabricated and fitted to increase the patient's OVD by 3.2 mm. During the one‐month wear period, the patient reported no discomfort or TMD‐related symptoms, indicating successful functional adaptation to the increased OVD. The patient also expressed satisfaction with the esthetic improvement provided by the splints, praising the comfort and functionality of FRED. The clinical experience and esthetic preview provided by the splints resulted in the patient's full acceptance of the proposed comprehensive rehabilitation treatment plan, paving the way for the subsequent restorative phase.

Written, informed consent was obtained from the patient.

## 4 Discussion

Advancements in technology have significantly enhanced the speed and effectiveness of dental treatments, particularly in integrating esthetic elements such as facial profiles and digital smile designs. While increased accuracy in data acquisition from novel digital tools has improved clinical workflows, it is essential to recognize that the quality of outcomes still heavily depends on clinical expertise and judgment rather than solely on digital advancements [11]. The diagnostic workflow described in this study serves a dual purpose: supporting functional assessment and offering esthetic previsualization. This approach is particularly valuable when the tool acts as a temporary prosthesis during the planning and testing phases of restorative treatments [12]. Furthermore, the tooth‐colored splint, designed to leverage the natural undercuts of existing teeth, offers a non‐invasive and reversible solution, emphasizing its role as a diagnostic and pre‐prosthetic aid.

The described technique addresses various clinical indications, providing flexibility in both diagnostic and therapeutic applications. Functional testing allows clinicians to evaluate jaw stability, while esthetic previsualization offers patients insights into potential cosmetic outcomes before committing to definitive treatments. Furthermore, FRED can serve as an interim solution for complex restorative cases and stabilize teeth affected by secondary occlusal trauma due to periodontitis [13, 14].

The modification of the OVD remains a nuanced aspect of restorative dentistry. Such changes require accurate diagnosis and meticulous planning to minimize risks and optimize outcomes [1]. Adjustments within a 5 mm range are generally well‐tolerated, with temporary discomfort resolving within 2 weeks [15]. The process typically involves balancing functional requirements and esthetic objectives, considering various clinical markers such as craniofacial alignment, patient comfort, and occlusal harmony.

In the traditional workflows, centric relation (CR) is often determined using techniques like bimanual manipulation or chin‐point guidance, sometimes with the aid of deprogramming devices [16, 17]. Fully digital workflows, however, offer alternatives by incorporating repeatable mandibular kinematics into virtual models to guide OVD adjustments [18, 19, 20]. In the present study, OVD adjustments were guided by the rest position, a widely adopted concept that reflects a naturally balanced and relaxed mandibular posture. Moreover, no deprogramming devices were used.

The rationale for using the rest position to determine a new OVD is based on the concept that physiological vertical dimension at rest (VDR) occurs within a comfort zone. The therapeutically established OVD is not a fixed reference point but rather a dynamic dimension within a zone of physiological tolerance [21, 22]. The latter is a range in terms of mm rather than a fixed position, in which the mandible is naturally positioned when at rest, with minimal tension in the jaw muscles [23]. In particular, VDR is defined as the postural position of the mandible when an individual is resting comfortably in an upright position and the associated muscles are in a state of minimal contraction [24]. The rest position of the mandible is a naturally maintained posture that the body typically assumes unconsciously, or voluntarily adopts when needed. As the rest position provides a naturally balanced and relaxed alignment of the jaw, changes in VDO that remain within this comfort range may be well tolerated and allow the patient to adapt without significant discomfort or functional disruption. This position is established through the interplay of three main mechanisms. First, postural muscle tonicity plays a significant role. Muscle tone, particularly in the temporalis muscle, is crucial for setting the resting position of the mandible. The temporalis muscles, as the most powerful elevators of the jaw and the muscles with the highest density of spindles, are primarily responsible for holding the mandible in this balanced state [25]. Second, the reflex mechanism governs the rest position by involving key components of the trigeminal nerve, specifically the mesencephalic and sensory nuclei. These centers receive sensory feedback from proprioceptors and exteroceptors about the position and movement of the jaw. In response to this input, the motor nucleus of the trigeminal nerve adjusts the mandible to maintain its resting position, based on the body's need for stability and balance [26]. Lastly, gravity and the elasticity of the surrounding soft tissues help stabilize the mandible against gravitational forces. The passive viscoelastic properties of perioral soft tissues, including muscles and ligaments, act to support the mandible's resting posture without continuous active muscle engagement, contributing to the ease and stability of this position [27].

Overall, the described dental technique requires access to advanced digital technology, which may increase the initial cost of treatment. Clinicians, together with dental technicians, should also be trained in using these tools effectively. Moreover, this technique may require CBCT with a large field of view. However, the integration of CBCT and 3D facial scanning is a significant advantage of this technique, ensuring that FRED is tailored not only to occlusal and functional needs but also to the patient's facial anatomy, promoting harmony between dental and esthetic outcomes [28]. The acquisition of a CBCT with a large field of view, using low radiation dosage, together with 4D technology, may allow clinicians to achieve an adjunctive diagnostic value, especially in cases needing a reorganizational approach [29]. Despite these considerations, the benefits of improved precision, functionality, and patient satisfaction may outweigh the limitations.

## 5 Conclusions

This technique integrates digital and traditional prosthetic principles into a patient‐centered, reversible, and adaptable workflow. It enables a precise, non‐invasive, and dynamic evaluation of occlusal vertical dimension modifications. By incorporating digital recording, facial scanning, and CAD/CAM fabrication, the approach enhances functional stability and esthetic visualization while minimizing patient discomfort. Future studies should investigate long‐term clinical outcomes and broader applications in complex prosthetic cases.

## Conflicts of Interest

The authors declare no conflicts of interest.

## Data Availability

Data sharing a not applicable to this article as no new data were created or analyzed in this study.
